# COVID-19-Related Challenges Among Patients With Hearing Loss Prescribed Hearing Aids, and Their Awareness of Telehealthcare

**DOI:** 10.7759/cureus.88324

**Published:** 2025-07-19

**Authors:** Ahmad M Alrasheed, Khalid T Ardi, Montasir Junaid, Omar Z Alaidaroos, Mona Alshehri, Amirah Abumismar, Salmah M Alharbi

**Affiliations:** 1 Otolaryngology - Head and Neck Surgery, Riyadh Third Health Cluster, Riyadh, SAU; 2 Otolaryngology - Head and Neck Surgery, King Faisal Medical City for Southern Regions, Abha, SAU; 3 Otolaryngology - Head and Neck Surgery, Armed Forces Hospital Southern Region, Khamis Mushait, SAU; 4 Otorhinolaryngology – Head and Neck, Audiology, and Speech Pathology, Mukhtar A. Sheikh Memorial Welfare Hospital, Multan, PAK; 5 Otolaryngology - Head and Neck Surgery, King Abdulaziz Hospital Jeddah, Jeddah, SAU; 6 Otolaryngology - Head and Neck Surgery, Asir Central Hospital, Abha, SAU; 7 Otolaryngology, Aseer Central Hospital, Abha, SAU; 8 Family Medicine, Family Medicine Academy, Qassim Health Cluster, Buraidah, SAU

**Keywords:** covid 19, face mask, hearing-aid, international outcome inventory - hearing aid, kingdom of saudi arabia (ksa), tele medicine

## Abstract

Background

The COVID-19 pandemic presented serious challenges in accessing health care, especially for individuals with hearing loss who rely heavily on both sight and sound. Communication and routine care, such as hearing aid management, were negatively impacted by the mandatory use of face masks and the limited availability of clinics.

Objective

This study aimed to assess the difficulties faced by patients with hearing loss who were prescribed hearing aids during the COVID-19 pandemic and to evaluate their awareness and acceptance of telehealthcare services.

Methods

A cross-sectional study was conducted at the Armed Forces Hospital, Southern Region in Aseer Province, Saudi Arabia. The sample included 210 individuals aged 18-65 years who had a medical diagnosis of hearing loss and were using conventional hearing aids prescribed between 2017 and 2019. SPSS version 22 was used for both descriptive and inferential statistical analyses.

Results

Among the 210 respondents, 109 (51.9%) reported difficulty communicating with individuals wearing face masks, primarily due to an inability to lip-read (n = 15, 7.3%) and muffled speech (n = 75, 35.8%). Despite this, 130 participants (61.9%) experienced no difficulty using their hearing aids while wearing a mask. Although 166 (80.2%) preferred in-person consultations over telehealth, 69 (32.8%) reported being less likely to visit a clinic due to the pandemic. Communication challenges were more prevalent among users with bilateral (n = 42, 20.0%) and severe hearing loss (n = 57, 27.1%). Statistically significant associations were found between hearing loss type and both preference for in-person visits (χ² = 6.01, p = 0.049) and mask-related communication difficulty (χ² = 12.37, p = .002).

Conclusion

The use of face masks and restricted clinic access during the COVID-19 pandemic significantly inconvenienced individuals using hearing aids. Although tele-audiology presents a promising alternative, uptake remains low due to limited patient awareness and acceptance. Investment in telehealth infrastructure and expanded patient education is essential to ensure continuity of hearing care during future public health emergencies.

## Introduction

The COVID-19 outbreak posed unprecedented challenges to healthcare delivery systems globally, especially for people with sensory impairments, including hearing loss [1]. Among the numerous public health precautions implemented to reduce the spread of the virus, the strict adoption of face masks and social distancing was found to greatly impair human-to-human verbal communication [2, 3]. These obstacles intensified the challenges faced by patients with hearing impairments, who rely heavily on facial expressions and lip-reading, especially in poor and noisy environments [4]. Studies revealed that face masks muffle high-frequency sounds used in speech perception and recognition, as well as obscure the visual elements of communication, thereby creating unique challenges for successfully using a hearing aid (HA) [3-7]. In addition to communication barriers, access to in-person services such as audiology and otolaryngology care was also disrupted by the pandemic [6]. Some patients could not be fitted with their HAs, have their devices adjusted, or receive follow-up services during the pandemic, as many audiologists in some countries, including Saudi Arabia, shut down their services [1, 8]. Patients who depended largely on routine consultations were among those whose hearing-related health was most affected by this interruption [9].

Tele-audiology, along with broader telehealth services, was among the innovations used to empower providers to ensure continued patient care amid access limitations [4, 10]. Nonetheless, patients were generally less aware of these services and embraced them to varying degrees. Although the experience with tele-audiology has been largely positive among audiologists, who particularly praise its benefits related to convenience and scheduling flexibility, many patients remain doubtful about its potential for treating hearing-related conditions [11-13]. This research is intended to investigate the conceptual challenges faced by patients with hearing loss who were prescribed hearing devices in Saudi Arabia during the COVID-19 era. It also explores patients’ understanding of tele-healthcare services and their preferences regarding in-person versus remote care. Through such considerations, the study aims to address how pandemic-related developments influenced the management of hearing loss, the usability of HAs under mask-wearing conditions, and the relationship between tele-audiology and a potentially sustainable healthcare model in the post-pandemic period.

## Materials and methods

Study design

A cross-sectional approach was employed in this study to ascertain the problems faced by users of HAs during the COVID-19 pandemic and their familiarity with tele-healthcare services.

Setting and timeframe of research

The study was carried out at the Armed Forces Hospital - Southern Region (AFHSR) in the Aseer region, Saudi Arabia. Military personnel, their families, and civilians receive free medical care at AFHSR, a tertiary public institution and one of the largest hospitals in the region. The hospital serves a population of approximately four million [14], covering the Aseer region along with neighboring areas. Patient interviews were conducted between August 2021 and May 2022.

Study population

The study population comprised adults between the ages of 18 and 65 who were diagnosed with hearing loss at AFHSR between 2017 and 2019 and were prescribed conventional HAs. The response rate was 58.1%, with 361 eligible individuals contacted and 210 patients expressing willingness to participate (Table 1). Inclusion criteria included a recorded hearing threshold based on pure-tone audiometry and a conventional HA prescription. Patients with implantable HAs or incomplete audiometric records were excluded.

**Table 1:** Eligibility criteria. AFHSR: Armed Forces Hospital – Southern Region.

Inclusion	Exclusion
Aged 18-65 years	Aged <18 or >65 years
Male and female	Ineligible for AFHSR services
Military personnel and civilians	Patients with surgically implanted hearing aids
Presented with a hearing loss complaint to AFHSR	Patients prescribed hearing aids for tinnitus masking only
Pure tone audiometry confirming conductive, sensorineural, or mixed hearing loss	Patients without pre-fitting pure tone audiometry results in their electronic file
Prescribed conventional hearing aids by an otolaryngologist between 2017-2019	-

Data variables

The characteristics of HA use (type, duration of use), as well as the type and degree of hearing loss, were collected using electronic medical records. Other data obtained during the interviews included general health status, comorbidities, otologic history, perceived barriers related to COVID-19, and preferences regarding tele-health services.

Data collection procedures

Information was collected through face-to-face or phone interviews, depending on the communication ability and personal preference of each participant. For individuals with pronounced hearing impairment, interviews were conducted via WhatsApp online chat to ensure accessibility. Prior to participation, the purpose of the questionnaires was thoroughly explained, and informed consent was obtained either verbally or via chat. Each interview lasted approximately 30 minutes.

Statistical analysis

After extraction, the data were edited, coded, and entered into IBM SPSS version 22 (IBM Corp., Armonk, NY). All statistical analyses were performed using two-tailed tests. A p-value of less than 0.05 was considered statistically significant. Descriptive analysis was conducted for participants' biodemographic information, hearing loss characteristics, HA use, clinical symptoms, and the impact of the COVID-19 pandemic on clinical consultations among HA users, using frequency and percentage distributions. The relationship between the type, degree, and side of hearing loss and the impact of COVID-19 on access to medical treatment was assessed using cross-tabulation. The Pearson chi-square test and the exact probability test (used for small frequency distributions) were applied to determine the significance of these associations.

## Results

Sample description

The total number of participants was 210. The participants ranged in age from 18 to 65 years, with a mean age of 48.8 ± 13.5 years. Of these, 115 participants (54.8%) were men, and 183 participants (87.1%) were married. A total of 126 (60%) had less than a high school education, whereas 21 (10%) held a university degree. In terms of chronic conditions, 112 (53.3%) had no chronic health issues, 52 (24.8%) had hypertension, 12 (5.7%) had hypothyroidism, and 65 (31%) had diabetes (Table 2).

**Table 2:** Bio-demographic data of study participants.

Bio-demographic data	No.	%
Age in years		
<40	50	23.80%
40-59	98	46.70%
60+	62	29.50%
Gender		
Male	115	54.80%
Female	95	45.20%
Marital status		
Married	183	87.10%
Not married	27	12.90%
Educational level		
Below high school	126	60.00%
Diploma / high school	63	30.00%
University / above	21	10.00%
Chronic diseases		
None	112	53.30%
Diabetes mellitus	65	31.00%
Hypertension	52	24.80%
Hypothyroidism	12	5.70%
Renal disease	4	1.90%
Asthma	1	0.50%
Depression	5	2.40%
Heart disease	2	1.00%
Others	7	3.30%

Hearing loss characteristics and HA use

Seventy-two (34.3%) and 57 (27.1%) of the participants had moderate and severe hearing loss, respectively. In 140 (72.2%) of the right-side cases, the cause was sensorineural hearing loss (SNHL). On the left side, 145 (73.6%) of the participants had SNHL, while 52 (24.8%) had severe hearing loss and 76 (36.2%) had moderate hearing loss. A total of 42 participants (20.0%) had bilateral hearing loss. The most commonly reported HAs were in-the-ear (ITE) in 43 cases (20.5%) and behind-the-ear (BTE) in 159 cases (75.7%). Of all participants, 163 (67.3%) reported awareness of their diagnosis, and 135 (64.3%) had been wearing HAs for more than one year (Table 3).

**Table 3:** Hearing loss and hearing aid data of research participants. CHL: Conductive Hearing Loss; SNHL: Sensorineural Hearing Loss.

Hearing loss and hearing aid data	No.	%
Right ear hearing loss degree		
Normal	16	7.60%
Mild	27	12.90%
Moderate	72	34.30%
Moderately severe	16	7.60%
Severe	57	27.10%
Profound	22	10.50%
Right ear hearing loss type		
CHL	17	8.80%
SNHL	140	72.20%
Mixed	37	19.10%
Left ear hearing loss degree		
Normal	13	6.20%
Mild	27	12.90%
Moderate	76	36.20%
Moderately severe	16	7.60%
Severe	52	24.80%
Profound	26	12.40%
Left ear hearing loss type		
CHL	16	8.10%
SNHL	145	73.60%
Mixed	36	18.30%
Ear for which hearing aid was used		
Right	93	44.30%
Left	75	35.70%
Bilateral	42	20.00%
Type of hearing aid used		
Behind-the-ear (BTE)	159	75.70%
In-the-ear (ITE)	43	20.50%
In-the-canal (ITC)	6	2.90%
Completely-in-canal (CIC)	2	1.00%
Duration of hearing aid use		
< 1 month	6	2.90%
3 months	13	6.20%
6 months	23	11.00%
1 year	33	15.70%
> 1 year	135	64.30%
Patient awareness of diagnosis for hearing aid prescription		
Yes	163	77.60%
No	10	4.80%
Not sure	37	17.60%

Clinical symptoms and risk factors

A total of 24 patients (11.4%) reported auditory trauma, 17 (8.1%) had a history of head trauma, and 5 (2.4%) reported both. In the two weeks prior to the patient interviews, the most common symptoms reported were: tinnitus in 60 (28.6%), vertigo/dizziness in 44 (21.0%), wax impaction while wearing HA in 37 (17.6%), pus ear discharge in 23 (11.0%), and new or worsening hearing loss in 84 (40.0%). Among those offered an alternative to HA, 33 (53.2%) received non-surgical options, and 29 (46.8%) were offered surgical alternatives. A total of 27 patients (12.9%) had undergone ear surgery. Regarding medical consultations, 76 (36.2%) attended the ENT/audiology clinic annually, whereas 88 (41.9%) never did so (Table 4).

**Table 4:** Risk factors, clinical symptoms, and past medical history data of research participants. ENT: Ear, Nose, Throat; HA: Hearing aid.

Past medical history data	Count	%
Exposure to any of		
Acoustic trauma	24	11.40%
Head trauma	17	8.10%
Both of them	5	2.40%
None	164	78.10%
Symptoms in the past 2 weeks		
None	52	24.80%
New worsening of hearing	84	40.00%
Tinnitus	60	28.60%
Vertigo/dizziness	44	21.00%
Wax impaction affecting hearing aid use	37	17.60%
Pus ear discharge	23	11.00%
Alternative treatment was offered		
Yes	62	29.50%
No	148	70.50%
Type of alternative treatment		
Non-surgical	33	53.20%
Surgical	29	46.80%
Previous ear surgeries		
Yes	27	12.90%
No	183	87.10%
Frequency of ENT/audiology clinic visit after HA prescription		
Never	88	41.90%
Every month	3	1.40%
Every 3 months	7	3.30%
Every 6 months	36	17.10%
Every year	76	36.20%

COVID-19-related challenges

Sixty-nine participants (32.8%) reported that they were less likely to visit the clinic for hearing problems due to the pandemic. While 130 participants (61.9%) reported no difficulty using masks with HAs, 109 (51.9%) experienced communication issues when interacting with individuals wearing masks. Among those reporting communication problems, 39 (35.8%) cited muffled voices, 8 (7.3%) reported an inability to lip-read, and 62 (56.9%) experienced both issues. Only 12 participants (5.8%) preferred online appointments, 29 (14.0%) had no preference, and 166 (80.2%) preferred in-person clinic visits, despite growing interest in tele-audiology. A total of 166 respondents (80.2%) stated they would prefer to physically visit the clinic if necessary during COVID-19 (Table 5).

**Table 5:** COVID-19-related consultation and communication responses among study participants. ENT: Ear, Nose, Throat.

Question / Response	No.	%
How likely are you to visit the ENT/audiology clinic if you have a problem with your hearing aids during COVID-19?		
Definitely will not go	19	9.00%
Mostly will not go	50	23.80%
Not sure	22	10.50%
Possibly would go	27	12.90%
Definitely would go	92	43.80%
Did you face problems wearing a face mask with your hearing aids during COVID-19?		
Not at all	130	61.90%
Faced some difficulties	25	11.90%
Not sure	46	21.90%
Faced significant difficulty	9	4.30%
Did you face any difficulty communicating with people who were wearing masks during COVID-19?		
Not at all	101	48.10%
Faced some difficulties	53	25.20%
Not sure	36	17.10%
Faced significant difficulty	20	9.50%
If yes, what were the reasons?		
I couldn't read their lips	8	7.30%
Voice sounded muffled	39	35.80%
Both	62	56.90%
During COVID-19, what was your preferred mode of attending the ENT/audiology clinic?		
Attend the clinic physically	166	80.20%
Attend the clinic online	12	5.80%
No preference	29	14.00%

The data demonstrated that 20 (27.8%) participants with mild/moderate hearing loss, 37 (35.2%) with severe hearing loss, and 12 (36.4%) with profound hearing loss were less likely to visit a clinic in case of a problem during COVID-19, whereas 41 (56.9%), 61 (58.1%), and 17 (51.5%), respectively, were more likely to attend. Concerning issues with mask use while wearing HAs, 24 (33.3%) of the mild/moderate group, 42 (40.0%) of the severe group, and 14 (42.4%) of the profound group reported difficulties, whereas 48 (66.7%), 63 (60.0%), and 19 (57.6%) reported no problems. Similarly, with communication while interacting with mask-wearing individuals, 40 (55.6%), 51 (48.6%), and 18 (54.5%) of the mild/moderate, severe, and profound hearing loss groups, respectively, reported difficulties, compared to 32 (44.4%), 54 (51.4%), and 15 (45.5%) who did not. Despite the pandemic, the majority of participants still favored face-to-face visits, with 57 (80.3%) preferring in-person visits compared to online consultations, which were preferred by only 1 (1.4%), 8 (7.7%), and 3 (9.4%) in the mild/moderate, severe, and profound groups, respectively. The results showed no significant differences between these groups on any variable (all p > 0.05), indicating relatively similar experiences and preferences regardless of the severity of hearing loss (Table 6).

**Table 6:** Effect of the COVID-19 pandemic on clinical consultation among persons using hearing aids according to their degree of hearing loss. ENT: Ear, nose, and throat; χ²: Chi-square value.

	Degree of Hearing Loss						p-value	X^2^
	Mild / moderate		Severe		Profound			
	No	%	No	%	No	%		
How possible is it to go to the ENT/audiology clinic if you have a problem with your hearing aids during COVID-19?							0.381	33.54
Less likelihood to go	20	27.8%	37	35.2%	12	36.4%		
Not sure	11	15.3%	7	6.7%	4	12.1%		
More likelihood to go	41	56.9%	61	58.1%	17	51.5%		
Did you face problems during COVID-19 with wearing a face mask with your hearing aids?							0.572	24.03
No difficulty at all	48	66.7%	63	60.0%	19	57.6%		
Faced difficulties	24	33.3%	42	40.0%	14	42.4%		
Did you face any difficulty communicating with people who were wearing masks during COVID-19?							0.624	14.91
No difficulty at all	32	44.4%	54	51.4%	15	45.5%		
Faced difficulties	40	55.6%	51	48.6%	18	54.5%		
During COVID-19, what is your preference on how to attend the ENT/audiology clinic?							0.543	13.77
Attend the clinic physically	57	80.3%	86	82.7%	23	71.9%		
Attend the clinic online	1	1.4%	8	7.7%	3	9.4%		
No preference	13	18.3%	10	9.6%	6	18.8%		

In analyzing the influence of COVID-19 by type of hearing loss, the likelihood of participants visiting the clinic did not significantly differ (p = .689). Among individuals with conductive hearing loss (CHL), 4 (26.7%) were unlikely to visit, 1 (6.7%) was unsure, and 10 (66.7%) were more likely to attend. A similar trend was observed in the SNHL group, where 44 (32.6%) were less likely, 17 (12.6%) were unsure, and 74 (54.8%) were more likely to go. In the mixed hearing loss group, 21 (35.0%) were less likely, 4 (6.7%) were unsure, and 35 (58.3%) were more likely to visit. Regarding mask use with HAs, there were notable differences (p = .002): 2 (13.3%) of CHL, 63 (46.7%) of SNHL, and 15 (25.0%) of mixed HL participants reported challenges. However, there was no significant difference in communication difficulties with mask-wearers (p = .527), with 7 (46.7%) of CHL, 74 (54.8%) of SNHL, and 28 (46.7%) of mixed HL participants reporting problems. Although the differences were not statistically significant (p = .120), the majority of participants in all groups preferred in-person appointments: 12 (80.0%) with CHL, 101 (75.9%) with SNHL, and 53 (89.8%) with mixed HL (Table 7).

**Table 7:** Effect of the COVID-19 pandemic on clinical consultation among persons using hearing aids according to the type of hearing loss. ENT: Ear, Nose, and Throat; CHL: Conductive Hearing Loss; SNHL: Sensorineural Hearing Loss; HL: Hearing Loss; χ²: Chi-square value.

	Type of Hearing Loss						p-value	X^2^
	CHL		SNHL		Mixed			
	No	%	No	%	No	%		
How possible it is to go to the ENT/Audiology clinic if you have a problem with your hearing aids during COVID-19?							0.689	6.57
Less likelihood to go	4	26.7%	44	32.6%	21	35.0%		
Not sure	1	6.7%	17	12.6%	4	6.7%		
More likelihood to go	10	66.7%	74	54.8%	35	58.3%		
Did you face problems during COVID-19 with wearing face mask with your Hearing Aids?							0.002	21.09
No difficulty at all	13	86.7%	72	53.3%	45	75.0%		
Faced difficulties	2	13.3%	63	46.7%	15	25.0%		
Did you face any difficulty communicating with people who are wearing masks during COVID-19?							0.527	5.71
No difficulty at all	8	53.3%	61	45.2%	32	53.3%		
Faced difficulties	7	46.7%	74	54.8%	28	46.7%		
During COVID-19, what is your preference on how to attend the ENT/Audiology Clinic?							0.120	14.06
Attend the clinic physically	12	80.0%	101	75.9%	53	89.8%		
Attend the clinic online	0	0.0%	8	6.0%	4	6.8%		
No preference	3	20.0%	24	18.0%	2	3.4%		

Analyzing the COVID-19 effect by the side of hearing loss showed no significant difference in participants’ willingness to visit the clinic (p = .875). Among those with right-sided hearing loss, 27 (29.0%) were less likely to attend, 11 (11.8%) were unsure, and 55 (59.1%) were more likely to go. In the left-sided group, 27 (36.0%) were less likely, 7 (9.3%) were unsure, and 41 (54.7%) were more likely to go. Among bilateral cases, 15 (35.7%) were less likely, 4 (9.5%) were unsure, and 23 (54.8%) were more likely to attend. However, significant differences were observed in experiences with mask use and communication. For mask-wearing difficulty while using HAs, 26 (28.0%) of right-sided, 34 (45.3%) of left-sided, and 20 (47.6%) of bilateral hearing loss participants reported problems (p = .026). Similarly, for communication with mask-wearers, 41 (44.1%) of right-sided, 36 (48.0%) of left-sided, and 32 (76.2%) of bilateral hearing loss participants reported difficulties (p = .002). Despite these challenges, preference for in-person visits remained high across all groups: 71 (78.0%) with right-sided, 61 (82.4%) with left-sided, and 34 (81.0%) with bilateral hearing loss preferred face-to-face consultations, with no significant difference observed (p = 0.700) (Table 8).

**Table 8:** Effect of the COVID-19 pandemic on clinical consultation among persons using hearing aids according to the side of hearing loss. ENT: Ear, nose, and throat; χ²: Chi-square value.

	Side of Hearing Loss						p-value	X^2^
	Right		Left		Bilateral			
	No	%	No	%	No	%		
How possible it is to go to the ENT/Audiology clinic if you have a problem with your hearing aids during COVID-19?							0.875	3.67
Less likely to go	27	29.0%	27	36.0%	15	35.7%		
Not sure	11	11.8%	7	9.3%	4	9.5%		
More likely to go	55	59.1%	41	54.7%	23	54.8%		
Did you face problems during COVID-19 with wearing face mask with your Hearing Aids?							0.026	14.49
No difficulty at all	67	72.0%	41	54.7%	22	52.4%		
Faced difficulties	26	28.0%	34	45.3%	20	47.6%		
Did you face any difficulty communicating with people who are wearing masks during COVID-19?							0.002	21.97
No difficulty at all	52	55.9%	39	52.0%	10	23.8%		
Faced difficulties	41	44.1%	36	48.0%	32	76.2%		
During COVID-19, what is your preference on how to attend the ENT/Audiology Clinic?							0.700	3.16
Attend the clinic physically	71	78.0%	61	82.4%	34	81.0%		
Attend the clinic online	7	7.7%	2	2.7%	3	7.1%		
No preference	13	14.3%	11	14.9%	5	11.9%		

## Discussion

During the COVID-19 pandemic, many HA users experienced challenges in communication and access to care. Among the 210 respondents, 109 (51.9%) reported difficulty communicating with individuals wearing face masks due to muffled voices (n = 75, 35.8%) and inability to lip-read (n = 15, 7.3%), while 130 (61.9%) stated that they had no issues communicating with mask-wearing individuals while using their HAs. Despite pandemic-related restrictions, 166 participants (80.2%) preferred face-to-face consultations over telehealth services. Only 12 (5.8%) opted for online consultations, whereas 29 (14.0%) were indifferent. Notably, 69 participants (32.8%) indicated they were less willing to visit a clinic during the pandemic, highlighting limited accessibility to available services. Bilateral HL (n = 32, 76.2%) was associated with a higher likelihood of communication difficulties compared to right-sided (n = 41, 44.1%) and left-sided (n = 36, 48.0%) HL (p = 0.002). Mask-related difficulties were also more prevalent among bilateral HA users (n = 20, 47.6%, p = 0.026). A stronger preference for in-person visits was linked to severe HL (n = 57, 27.1%, p = 0.049) and mixed HL (n = 53, 25.2%, p = 0.049). These findings align with previous studies indicating that face masks not only obscure facial expressions and lip movements but also attenuate high-frequency sounds essential for speech intelligibility [3, 15, 16].

According to Benítez-Robaina S et al. [1], speech discrimination significantly decreased among HA users when communicating with individuals wearing surgical masks in noisy environments. The study emphasized that people with hearing impairments rely heavily on both auditory and visual cues, and the absence of either can disproportionately hinder comprehension. Additionally, a randomized controlled trial by [17] found that face masks negatively affected empathy and relational continuity in healthcare, suggesting that communication challenges extend beyond auditory limitations and into psychosocial domains. Some participants also reported physical discomfort or interference when wearing both masks and HAs simultaneously [18].

Although tele-audiology emerged as a viable solution for service continuity during lockdowns, the majority of our study participants preferred in-person consultations. This may be attributed to limited exposure, low digital literacy, or mistrust in the effectiveness of remote care. Prior studies have noted a mismatch between provider enthusiasm and patient acceptance of telehealth in audiology. A review study [19] also reported that face mask usage, social distancing, e-learning, and virtual communication during the pandemic created additional difficulties for individuals with HL. These issues contributed to adverse effects such as increased isolation and loneliness, restricted access to education and rehabilitation services due to school and center closures, and challenges in home-based online learning.

This discrepancy is important because tele-audiology can help reduce access disparities, especially among rural or mobility-limited populations [20]. However, to realize these benefits, greater efforts are needed to raise patient awareness, build confidence, and ensure access to the required technology [21]. Acceptance and usability of tele-audiology services may be enhanced through patient education and the development of simplified, user-friendly platforms, particularly in situations where face-to-face visits are unsafe or impractical [22].

The implications of these findings are significant in the context of future health crises and the development of resilient hearing healthcare systems. While face-to-face consultations remain the preferred option for many patients, the pandemic has highlighted the need for flexible and adaptive models of care. Incorporating acoustically transparent mask designs, deploying communication strategies that compensate for the loss of visual cues (e.g., transparent masks), and integrating tele-audiology are all approaches that could improve user experience and outcomes for HA users [23, 24]. Furthermore, training both providers and patients to use remote care toolkits should be a policy priority to ensure continuity of care during future disruptions.

There are various limitations associated with this study. The first potential limitation of the current study is its cross-sectional nature, as it imposes a constraint on the ability to establish a causal relationship between the difficulties experienced during the COVID-19 pandemic and the use or satisfaction of HAs. Second, the study relied on self-reported data, which may be subject to recall bias or may include social desirability bias. The sample also involved only those patients who were at a specific tertiary care center in Saudi Arabia; this is also likely to affect the outcome of the study in terms of its generalizability. Despite such limitations, the current research study has several strengths, such as a comparatively large sample size. Further, this investigational paper provides factual evidence of real-life practice and the internal realities of individuals using HA devices during the pandemic, an experience that is currently underrepresented in the literature. Future studies must consider the inclusion of longitudinal study designs to determine the impact of time and examine diverse populations in various locations to increase the applicability of the research. A recommendation has also been made for future studies to consider how patients can be involved in tele-audiology and whether clear face masks or other assistive communication tools are effective in practice.

## Conclusions

The COVID-19 pandemic posed significant challenges for individuals with HL, particularly those using HAs. The use of face masks disrupted information processing, and communication difficulties compounded the impairment by affecting sound quality and obscuring visual cues. Tele-audiology had the potential to address access issues; however, most patients preferred in-person visits when given the choice and had limited familiarity or confidence with remote care options. This disparity can be addressed through both infrastructural improvements and awareness-building efforts, not only to strengthen telehealth infrastructure but also to enhance patient education and trust in remote hearing services, ensuring continuity of care during future public health crises.
